# Therapy for Pulmonary Arterial Hypertension: Glance on Nitric Oxide Pathway

**DOI:** 10.3389/fphar.2021.767002

**Published:** 2021-11-12

**Authors:** Abraham Tettey, Yujie Jiang, Xiaohui Li, Ying Li

**Affiliations:** ^1^ Department of Pharmacology, School of Pharmaceutical Science, Central South University, Changsha, China; ^2^ Department of Health Management, The Third Xiangya Hospital, Central South University, Changsha, China; ^3^ Hunan Key Laboratory for Bioanalysis of Complex Matrix Samples, Changsha, China

**Keywords:** nitric oxide, pulmonary arterial hypertension, phosphodiesterase 5 inhibitor, proliferation, crosstalk

## Abstract

Pulmonary arterial hypertension (PAH) is a severe disease with a resultant increase of the mean pulmonary arterial pressure, right ventricular hypertrophy and eventual death. Research in recent years has produced various therapeutic options for its clinical management but the high mortality even under treatment remains a big challenge attributed to the complex pathophysiology. Studies from clinical and non-clinical experiments have revealed that the nitric oxide (NO) pathway is one of the key pathways underlying the pathophysiology of PAH. Many of the essential drugs used in the management of PAH act on this pathway highlighting its significant role in PAH. Meanwhile, several novel compounds targeting on NO pathway exhibits great potential to become future therapy medications. Furthermore, the NO pathway is found to interact with other crucial pathways. Understanding such interactions could be helpful in the discovery of new drug that provide better clinical outcomes.

## Introduction

Pulmonary arterial hypertension (PAH) is a fatal disease characterized by an increase in pulmonary arterial pressure with subsequent right ventricular failure and death (Rosenkranz, 2015). Generally, the major pathological changes of PAH are vasoconstriction and vascular remodelling of the small pulmonary arteries (Tuder et al., 2009) involving the thickening of the intima-media, smooth muscle cell proliferation, endothelial cell proliferative lesions formation, vascular inflammation and immune dysregulation (Tuder et al., 2007; Price et al., 2012; Humbert et al., 2019). There are different categories in PAH including idiopathic PAH, heritable PAH, drug or toxin-induced PAH and PAH associated with connective tissue disease, human immunodeficiency virus infection, portal hypertension, congenital heart disease and schistosomiasis (Galiè et al., 2016). Idiopathic PAH is the most common type of PAH in western countries with congenital heart disease-related PAH being the most prevalent in Asia (Humbert et al., 2006; Lim et al., 2019).

Currently approved PAH drugs such as prostacyclin analogues, endothelin receptor antagonists and phosphodiesterase-5 (PDE-5) inhibitors aim to control pulmonary vascular tone (Olsson and Hoeper, 2009). Enormous progress has been made in the therapeutic practice of PAH in the past decades but there is still a long way to go because none of these present drugs are curative. They reduce morbidity and only slightly improve survival with no effect on PAH mortality rate.

Many molecular pathways have been linked to the development and treatment of PAH.It has been revealed that the nitric oxide (NO) pathway plays a very essential and central role in regulating vascular tone in the pulmonary circulatory system by interacting with other crucial signaling pathways. Phosphodiesterase-5 (PDE-5) inhibitors and soluble guanylate cyclase stimulators are the current drug classes acting on this pathway. Meanwhile, novel compounds targeting on NO pathway exhibits great potential in development of next generation therapy medications. This review seeks to summarize the research progress of PAH regarding to NO pathway, discuss the critical role of NO pathway in understanding the pathophysiology of PAH and new drug development.

## Current Clinical Therapy in Pulmonary Arterial Hypertension

Current PAH therapy focuses on three main pathways. These are the prostacyclin, nitric oxide, and endothelin pathways. Drugs acting on these pathways are summarized in Table 1.

**TABLE 1:** Classification of drugs used in treating PAH.

Class	Generic name	Dosing	Side-effects
Prostacyclin analogs	Epoprostenol	Continuous infusion	Abdominal pain, anxiety, arrhythmias, arthralgia, chest discomfort, diarrhea
	Treprostinil	Continuous IV or SC, inhalation, Oral	Infusion site reaction, pain, headache, nausea, diarrhoea, vasodilation, jaw pain, rash
	Iloprost	Inhalation	Chest discomfort, cough, diarrhea, dizziness, dyspnoea, hemorrhage, headache, hypotension, nausea
Prostacyclin IP receptor agonist	Selexipag	Oral	Abdominal pain, anemia, appetite decreased, arthralgia, diarrhea, flushing, headache
Endothelin Receptor Antagonists	Bosentan	Oral	Anemia, diarrhea, flushing, gastroesophageal reflux disease, headache, nasal congestion, palpitations
	Ambrisentan	Oral	Abdominal pain, anemia, asthenia, constipation, dizziness, epistaxis, flushing, headaches, hearing impairment
	Macitentan	Oral	Anaemia, headache, increased risk of infection, nasal congestion
PDE5 Inhibitors	Sildenafil	Oral, Intravenous	Dry mouth, flushing, gastrointestinal discomfort, hemorrhage, myalgia, headache
	Tadalafil	Oral	Flushing, gastrointestinal discomfort, headaches, myalgia, nasal congestion, pain
Soluble Guanylate Cyclase Stimulator	Riociguat	Oral	Anaemia, constipation, diarrhoea, dizziness, dysphagia, gastroenteritis, gastrointestinal discomfort

### Prostanoids

Prostacyclin or prostanoid is produced in the endothelium and it is a potent vasodilator in the pulmonary vasculature (Gryglewski, 2008). Prostacyclin activate the prostacyclin receptor which leads to an increase in cyclic adenosine monophosphate (cAMP) production and consequently, vasodilatation (Coleman et al., 1994). Activating the IP receptor also leads to antithrombotic and antiproliferative effects in the pulmonary vasculature (Wharton et al., 2000; Vane and Corin, 2003). Examples of prostacyclins used in PAH management include epoprostenol and treprostinil. Selexipag is a non-prostanoid IP receptor agonist. Epoprostenol is the only drug shown to improve mortality (Barst et al., 2009) and remains the drug of choice in severe cases (Galiè et al., 2016). It is administered by continuous infusion. The different prostanoids have different routes of administration with varying degrees of efficacy.

### Endothelin-1 Receptor Blockers

Endothelin-1 is a potent vasoconstrictor produced by the endothelial cells and it facilitates pulmonary artery smooth muscle cell proliferation (Yanagisawa et al., 1988; Davie et al., 2002). Endothelin-1 binds to two main receptors namely endothelin receptor A (ET_A_) and endothelin receptor B (ET_B_). ET_A_ is predominant in vascular smooth muscle cells (VSMC) and facilitates contraction and proliferation of VSMCs in PAH (Rubin et al., 2011). ET_B_ is predominantly expressed in vascular endothelial cells where it enhances vasodilation via the production of prostacyclin and nitric oxide (NO) as well as clearance of ET-1 (Eguchi et al., 1993; Hirata et al., 1993; Seo et al., 1994). Interestingly, ET_B_ is also found in VSMCs where it possesses vasoconstrictive and proliferative properties (Seo et al., 1994). Endothelin-1 receptor blockers prevent endothelin-1 from binding to its receptors thereby abrogating its destructive effects in PAH. Endothelin-1 receptor blockers can be grouped as selective (eg., ambrisentan) and non-selective (eg., Bosentan and macitentan) depending on their endothelin-1 receptor binding properties (Correale et al., 2013). Bosentan is the first orally administered PAH drug, notably it causes abnormal liver function in some patients necessitating monthly liver function tests (Humbert et al., 2007). Ambrisentan and macitentan have lower chances of causing liver damage (Galiè et al., 2005a; Pulido et al., 2013).

### Drugs Acting on the Nitric Oxide Pathway

Nitric oxide (NO) is produced by the endothelial cells and serves as a potent vasodilator of the pulmonary circulation through cyclic guanosine monophosphate (cGMP). Phosphodiesterase-5 (PDE5) inhibitors such as sildenafil and tadalafil and the soluble guanylate cyclase stimulator, riociguat act on this pathway. Further explanation of drugs acting on this pathway is in section 4 of this review.

## Nitric Oxide Pathway and Its Implication in Pulmonary Arterial Hypertension

Nitric oxide (NO) is a biological molecule that regulates many physiological and pathological processes in the body. It was proposed that the release of a vasodilating factor by the endothelial cells as one of the mechanisms behind acetylcholine-induced vasodilation *in vivo* (Furchgott and Zarwodski, 1980)*.* The identity of nitric oxide as the endothelial-derived relaxing factor was not known until 1987 when two different studies confirmed it (Ignarro et al., 1987; Palmer et al., 1987).

**FIGURE 1:**
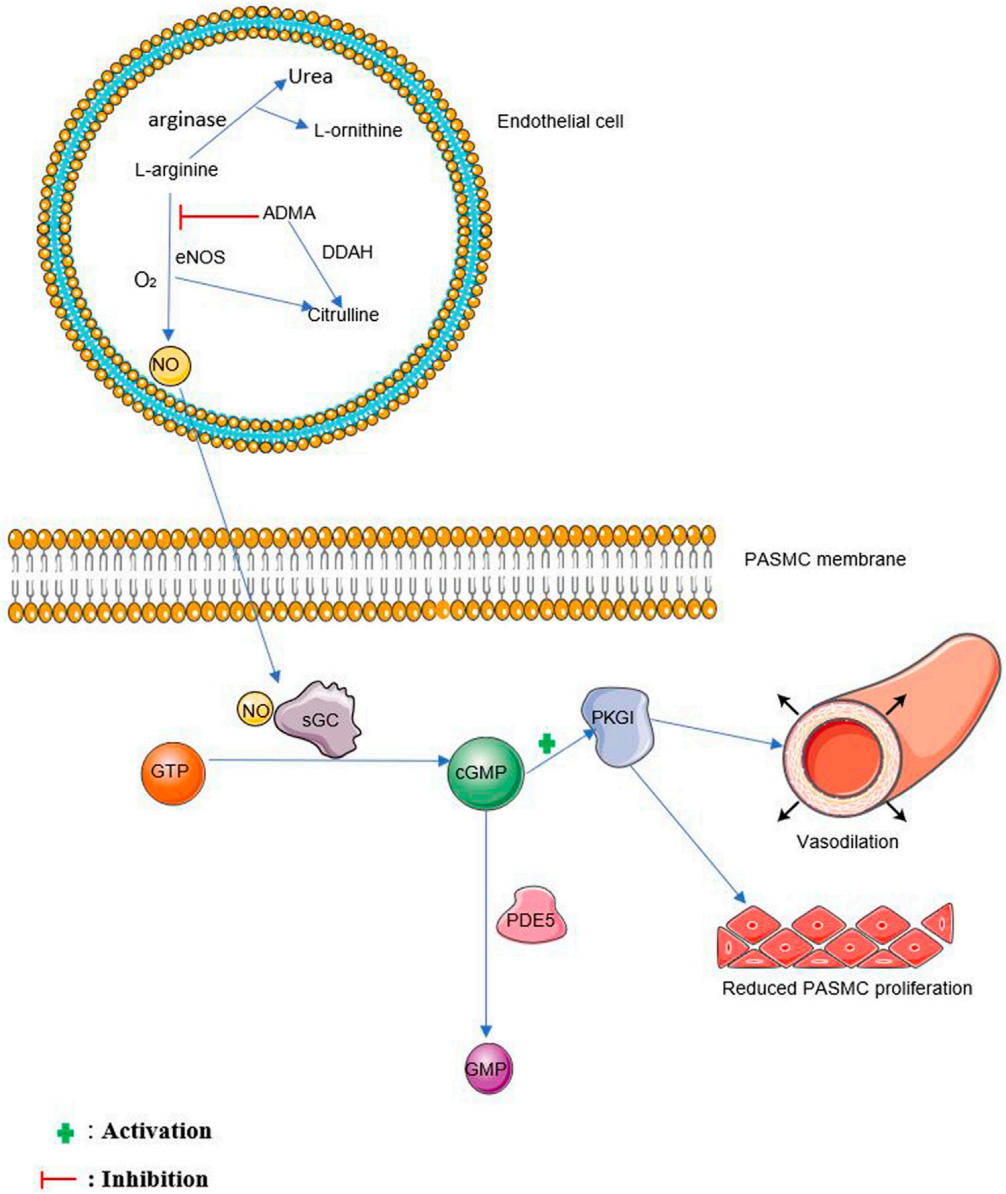
The NO pathway. NO, nitric oxide, sGC, soluble guanylate cyclase, GTP, guanosine triphosphate, cGMP, cyclic guanosine monophosphate, PDE5, phosphodiesterase 5, GMP, guanosine monophosphate, PKGI, protein kinase G I. eNOS, endothelial nitric oxide synthase, O_2_, oxygen, ADMA, Asymmetric dimethylarginine, DDAH, dimethylarginine dimethylaminohydrolase, PASMC, pulmonary arterial smooth muscle cell.

Nitric oxide is formed from the oxidation of L-arginine to form citrulline and NO in the presence of nitric oxide synthases (NOS) and molecular oxygen (Moncada and Higgs, 1993) as shown in Figure 1. Arginase competes with eNOS for L-arginine by converting L-arginine to urea and L-ornithine (Luiking et al., 2012). *N* ^*G*^ *, N* ^*G*^-dimethyl-L-arginine (ADMA) is known to inhibit this process by competing with L-arginine for eNOS (Zakrzewicz and Eickelberg, 2009). *N* ^G^, *N* ^G^-dimethylarginine dimethylaminohydrolases (DDAH1 and DDAH2) are responsible for the degradation of ADMA (Tain and Hsu, 2017). Three isoforms of nitric oxide synthases have been described in mammals. These are neuronal NOS (nNOS, NOS1), inducible NOS (iNOS, NOS2), and endothelial NOS (eNOS, NOS3) (Nathan and Xie, 1994).

Endothelium-derived NO diffuses into vascular smooth muscle cells and stimulates soluble guanylate cyclase (sGC) to produce cGMP, further activating associated protein kinases such as protein kinase G I (PKGI) which causes vasorelaxation (Sausbier et al., 2000; Surks, 2007). cGMP is broken down mainly by phosphodiesterase 5 (PDE5) (Francis et al., 2000). See Figure 1 for more details.

Conflicting levels of exhaled NO have been reported in PAH patients. Data from studies with patients suffering from idiopathic PAH and scleroderma-/drug-associated PAH indicated a reduction in exhaled NO as compared to healthy patients (Kharitonov et al., 1997; Kaneko et al., 1998; Archer et al., 2012). Other studies also found no significant change in exhaled NO levels in idiopathic PAH and scleroderma-associated PAH when compared to healthy subjects (Riley et al., 1997; Olivieri et al., 2006; Malekmohammad et al., 2019). There was no difference in exhaled NO after 3 months of treatment with endothelin receptor antagonists, guanylate cyclase stimulants, phosphodiesterase type 5 (PDE5) inhibitors and prostanoids in comparison with healthy subjects. However, other diagnostic markers such as 6MWD and N-terminal prohormone of brain natriuretic peptide (NT-proBNP) were significantly correlated to disease severity and treatment response (Malekmohammad et al., 2019). Confounding factors such as exhalation flow rate, measurement technique, the NO analyzer used, nasal NO contamination, age, height and smoking (Borrill et al., 2006; Dweik et al., 2012) makes exhaled NO an unreliable marker that needs standardization to be of diagnostic value in PAH.

The use of NO Plasma metabolites (NO_X_) as a biomarker and prognostic indicator of PAH is being studied. A study found that patients (age range: 20–57 years) with IPAH had reduced levels of plasma NOx, which correlated inversely with mPAP and patient survival (Zhang et al., 2016). Contradictory studies found elevated levels of NOx in IPAH and congenital heart disease-associated PAH patients with age ranges of 31–77 and 5 days -12 years (Ikemoto et al., 2002; Malinovschi et al., 2011). The patients in the extreme age groups (age averages of 6 and 54 years) seem to show increased levels of NOx. The different results from the various studies could be attributed to the PAH type and ages of the patients studied (Zhang et al., 2016).

Reduced eNOS levels have been reported in PAH patients (Giaid and Saleh, 1995). Another study found an increased expression of eNOS in the plexiform lesions of PAH patients (Berger et al., 2012). The eNOS that is elevated in some PAH patients is likely to be in the uncoupled state causing it to produce more superoxides (NO scavengers) than NO (Klinger et al., 2013). eNOS uncoupling can occur as a result of a decrease in amounts of tetrahydrobiopterin (BH4), a cofactor for eNOS in NO synthesis. There are studies to confirm eNOS uncoupling in pulmonary hypertension models that are BH4-deficient (Khoo et al., 2005; Nandi et al., 2005).

Also, several studies have described elevated levels of ADMA in plasma and serum of IPAH and connective tissue disease (CTD)-associated pulmonary arterial hypertension patients (Kielstein et al., 2005; Fang et al., 2015; Liu et al., 2019). Monocotaline- and hypoxia-induced PAH rat model have been found to have increased ADMA levels with a corresponding decrease in DDAH levels (Millatt et al., 2003; Li et al., 2010). This makes ADMA/DDAH possible diagnostic indicators in PAH

NO signaling remains a very relevant pathway in understanding and treating PAH. Upregulation of NO signaling is crucial in the management of PAH.

## Crosstalk With Nitric Oxide

**FIGURE 2:**
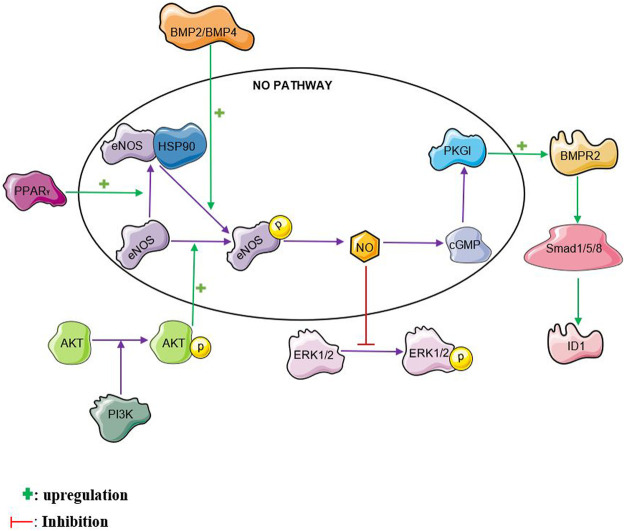
NO crosstalk. NO, nitric oxide, BMPR2, bone morphogenetic protein receptor 2 PPAR_γ_, peroxisome proliferator-activated receptor gamma ERK, extracellular signal-regulated kinase, PI3K, phosphatidylinositol 3-kinase, HSP90, heat shock protein 90, BMP2, bone morphogenetic protein 2, BMP4, bone morphogenetic protein 4, ID1, inhibitor of DNA binding 1

The nitric oxide pathway interacts with several other pathways reiterating its complex role in PAH. Nitric oxide interaction with some of these pathways is illustrated in Figure 2. The nitric oxide pathway has been shown to interact with the bone morphogenetic protein (BMP) pathway. Mutations in the bone morphogenetic protein receptor 2 (BMPR2) gene possess the strongest risk factor in the development of PAH, particularly heritable PAH (Machado et al., 2006). Bone Morphogenetic Protein Receptor II (BMPRII) and its ligands, BMP2 and BMP4 were found to be mediators of endothelial nitric oxide synthase activation (Gangopahyay et al., 2011). BMP2 and BMP4 activates eNOS by enhancing its phosphorylation by protein kinase A in pulmonary arterial endothelial cells (PAEC). Interestingly, knocking down BMPRII in PAEC reduced the ability of BMP2 and BMP4 to stimulate eNOS phosphorylation suggesting a crucial role of BMPRII in eNOS activation (Gangopahyay et al., 2011). Plasma nitric oxide metabolites have also been found to be markedly reduced in idiopathic pulmonary arterial hypertension patients with BMPR2 mutations (Zhang et al., 2016).Protein Kinase G I (PKGI) which is one of the downstream targets in the nitric oxide pathway has been found to crosstalk with the BMP pathway. PKGI contributes to the activation of BMP signaling by BMP4 in Human PASMCs (Yang et al., 2013). PKGI upregulates BMP signaling by phosphorylating the BMPRII receptor and smad proteins. Furthermore, PKGI binds to the phosphorylated smad proteins to form a complex and translocates into the nucleus where it upregulates the transcription of Id1 mRNA (Schwappacher et al., 2013). This then facilitates apoptosis and inhibits PASMC proliferation (Schwappacher et al., 2013; Yang et al., 2013).

The extracellular signal-regulated kinase 1/2 (ERK 1/2) pathway plays a significant role in the pathogenesis of PAH. Activation of the ERK pathway is known to be associated with increased proliferation and reduced apoptosis of PASMCs (Yang et al., 2008; Zhou et al., 2019). NO has been found to induce S-nitrosylation of ERK1/2 leading to reduced ERK phosphorylation and improved MCF-7 cell apoptosis (Feng et al., 2013). This crosstalk could be one of the ways with which NO inhibits proliferation and enhances apoptosis in PASMCs. S-nitrosylation of ERK1/2 by NO in PASMCs is yet to be explored.

The phosphatidylinositol 3-kinase/protein kinase B (PI3K/Akt) pathway is another major pathway that is implicated PAH. There have been contradictory reports on its role in PAH (Fang et al., 2016; Huang et al., 2017; Zheng et al., 2017). The PI3K/Akt pathway is known to phosphorylate eNOS and increase NO levels in many cell types including human pulmonary arterial endothelial cells (HPAECs) (Kim et al., 2008; Liang et al., 2009; Li et al., 2014). This helps to attenuate monocrotaline-induced pulmonary artery endothelial dysfunction and PAH in rats (Li et al., 2014; Zheng et al., 2017). Relaxation of rat pulmonary artery rings is enhanced through the activation of eNOS by the PI3K/Akt pathway (Zhang et al., 2010).

There is evidence that Peroxisome proliferator-activated receptor gamma (PPARγ) activity is reduced in PASMCs from idiopathic PAH patients (Falcetti et al., 2012). Activation of the Peroxisome proliferator-activated receptor gamma (PPARγ) pathway is known to reduce monocrotaline-and hypoxia-induced PAH in rats (Liu et al., 2012; Legchenko et al., 2018). Increased PPARγ expression has been found to enhance the phosphorylation and activation of eNOS in human pulmonary arterial endothelial cells (Li et al., 2014). Enhanced interaction between heat shock protein 90 and eNOS is one of the ways with which PPARγ facilitates eNOS phosphorylation. PPARγ agonists such as rosiglitazone, ciglitazone and pioglitazone have been found to stimulate eNOS activation through various mechanisms. Rosiglitazone was found to activate eNOS by stimulating heat shock protein (HSP)-90–eNOS interaction, with a subsequent eNOS phosphorylation at Ser^1177^ (Polikandriotis et al., 2005). Ciglitazone also caused a reduction in human umbilical vein endothelial cells (HUVEC) membrane NADPH-dependent superoxide anion generation leading to a decrease in endothelial superoxide anion generation and oxidative stress with a resulting increase in NO bioavailability (Hwang et al., 2005; Polikandriotis et al., 2005). Rho-kinase, a serine–threonine kinase is known to cause endothelial dysfunction by inactivating eNOS and reducing NO generation (Nohria et al., 2006). Pioglitazone enhances the dephosphorylation on tyrosine residues of the Vav protein, a group of guanosine nucleotide exchange factors (GEFs) involved in the activation of Rho Kinase. The deactivation of Rho Kinase leads to an increase in eNOS activation with a consequent increase in NO production (Wakino et al., 2004). The PPARγ agonist, telmisartan was found to increase the expression of DDAH II thereby enhancing the degradation of ADMA and increasing NO levels in human umbilical vein endothelial cells (Scalera et al., 2008).

## Current Drugs ON Nitric Oxide Pathway in Pulmonary Arterial Hypertension

There are two groups of drugs currently used in PAH that act on the nitric oxide pathway, the phosphodiesterase 5 inhibitors (sildenafil and tadalafil) and soluble guanylate cyclase inhibitors (riociguat).

### Phosphodiesterase-5 Inhibitors

As the name suggests, PDE5 inhibitors inhibit the PDE5 enzyme responsible for the breakdown of cGMP. This increases the levels of cGMP which activates protein kinase G and consequently leads to vasodilation. PDE5 is very abundant in smooth muscle cells (Rybalkin et al., 2003). Sildenafil was the first PDE5 inhibitor accepted for the management of PAH.

The SUPER-1 trial found 20, 40 and 80 mg doses of sildenafil to increase 6MWD by 45, 46, and 50 m respectively after 12 weeks. These doses also reduced mPAP by 2.1, 2.6 and 4.7 mmHg respectively. The was no significant reduction in clinical worsening (as measured by death, hospitalization for pulmonary hypertension, initiation of prostacyclin and initiation of bosentan) in the different sildenafil dose groups when compared to the placebo. Also, there were no significant dose-dependent changes in exercise capacity making the 20 mg dose the most appropriate for clinical use (Galiè et al., 2005b). A follow-up open-label trial by the SUPER-2 group found the improvement in 6MWD to be maintained after 1 year with a value of 51 m (Rubin et al., 2011). Sildenafil caused mild side effects such as headaches, flushing, and dyspepsia.

Contrary to the SUPER-1 study, the PACES-1 study found the combination of sildenafil with long-term intravenous epoprostenol therapy to improve clinical worsening as compared to intravenous epoprostenol alone after 16 weeks (Simonneau et al., 2008). The proportion of patients that needed a change in epoprostenol dose (an indicator of clinical worsening) was 0.195 in the epoprostenol-only group with a lower proportion (0.062) in the combination group. The sildenafil dose for this study was titrated up from 20 to 40 mg and 80 mg three times daily over 16 weeks depending on patient drug tolerability. An open-label extension study of the PACES-1 trial found the long-term combination of sildenafil and intravenous epoprostenol to be well tolerated with 33% of patients have a maintained or improved 6MWD after 3 years(Simonneau et al., 2014).

Administration of bosentan (125 mg twice daily) to patients on stable sildenafil therapy resulted in no significant difference between the two groups with regards to the primary endpoint (time to the first morbidity/mortality event) after 16 weeks in an event-driven trial. This may be due to limitations of the study such as enrollment of patients with many comorbidities and a high rate of patient dropout, to name but a few. Nonetheless, the addition of bosentan yielded an increase in 6MWD by 7.2 ± 66.0 m in the bosentan group with a reduction of 14.6 ± 80.4 m in the placebo group after 16 weeks. The mean difference in 6MWD between the two groups was 21.8 m. However, this was only a secondary and exploratory endpoint of the study (McLaughlin et al., 2015).

Conversely, a 12 weeks randomized controlled clinical trial found the administration of sildenafil (20 mg three times daily) to patients on stable bosentan therapy offers no significant benefit in terms of the 6MWD as compared to bosentan alone (Vizza et al., 2017). Limitations such as a possible ceiling effect in patients receiving effective bosentan therapy could be responsible for the insignificant difference between the groups.

In a 16 weeks double-blind, placebo-controlled study, 40 mg once daily administration of tadalafil was found to increase 6MWD by 44 m in the bosentan-naive group and by 23 m in patients on bosentan background therapy. These increments were statistically significant in comparison to the placebo group. Tadalafil also reduced the time to clinical worsening and the incidence of clinical worsening (68% relative risk reduction). The most common side-effects were headache, myalgia, and flushing (Galiè et al., 2009a). A 52 weeks extension study confirmed tadalafil to be well-tolerated with a sustained improvement in the 6MWD (Oudiz et al., 2012).

As compared to tadalafil and ambrisentan monotherapies, the combination of tadalafil and ambrisentan resulted in fewer occurrences of clinical failure as defined by the first occurrence of a composite endpoint of death, hospitalization for worsening pulmonary arterial hypertension, disease progression, or unsatisfactory long-term clinical response. Clinical failure occurred in only 18% of patients in the combination group with 34 and 28% occurrences in the ambrisentan only and tadalafil only groups respectively. Side-effects such as headache and nasal congestion occurred more in the combination group than in the monotherapy groups (Galiè et al., 2015a). Because of moderate quality evidence, the 2019 CHEST guideline weakly recommends the initial combination therapy with ambrisentan and tadalafil to improve 6MWD for treatment-naive PAH patients with WHO FC II and III. Also, the addition of tadalafil to improve 6MWD of stable or symptomatic PAH patients on background therapy with ambrisentan is weakly recommended because of low-quality evidence (Klinger et al., 2019).

Vardenafil is also a PDE5 inhibitor. A randomized, double-blind, placebo-controlled study found it to be well tolerated in patients with PAH and also increase 6-MWD by 69 m at a dose of 5 mg twice daily. It also reduced mean pulmonary arterial pressure by 5.3 mmHg at 12 weeks (Jing et al., 2011). Vardenafil was found to significantly decrease mean pulmonary arterial pressure when used for acute vasoreactivity testing in patients with PH (Sandqvist et al., 2015). Vardenafil is not yet approved for the treatment of PAH.

### Soluble Guanylate Cyclase Stimulators

Soluble guanylate cyclase is activated by NO to convert guanosine triphosphate (GTP) to cGMP (Stasch et al., 2001). sGC stimulators directly activate soluble guanylate cyclase in smooth muscle cells. sGC stimulators also stabilize the complex formed between NO and sGC to enhance cGMP production (Stasch et al., 2011). Riociguat is the only approved sGC stimulator for treating PAH. It is approved to be used up to a maximum dose of 2.5 mg three times daily. Riociguat increases 6-MWD by a mean of 36 m as compared to placebo after 12 weeks. It also significantly decreases mPAP by 9 ± 11 mmHg from baseline. In the RESPITE trial, riociguat proved to be a viable alternative for patients with PAH not responding to PDE5 inhibitors because of reduced endogenous NO production (Hoeper et al., 2017a). The prospective randomized REPLACE trial further confirmed that patients irresponsive to PDE5 inhibitors are more likely to show clinical improvement upon switching to riociguat therapy (Hoeper et al., 2017b). This has made riociguat a crucial PAH treatment option. Some of its side effects include headache, syncope, and hypotension (Ghofrani et al., 2013a). There was sustained improvement in exercise capacity after 1 year as observed in a long-term extension trial (Rubin et al., 2011). Riociguat is also approved for treating inoperable chronic thromboembolic pulmonary hypertension (Ghofrani et al., 2013b). The combination of riociguat and PDE5 inhibitors is contraindicated due to increased hypotension (Galiè et al., 2015b).

### Inhaled Nitric Oxide

Even though inhaled NO has the potential for use in treating PAH, it is mostly used in acute vasoreactivity testing during right heart catheterization. This is to identify acute responders likely to benefit from calcium-channel blockers (CCBs). Acute responders are patients who show a decrease in mPAP of at least 10 mmHg to an absolute level below 40 mmHg with sustained or increased cardiac output (Badesch et al., 2009). These patients are rare with most of them having idiopathic PAH. Such patients have a very good prognosis with CCB use (Rich et al., 2010). Inhaled NO is only approved for the treatment of severe persistent pulmonary hypertension of the newborn (PPHN). There are not enough studies to support the safety and efficacy of the long-term ambulatory use of inhaled NO in PAH. Also, the lack of portable delivery systems and the need for continuous inhalation makes its daily ambulatory use impractical.

## Ongoing Pharmaceutical Research ON Nitric Oxide Pathway and Its Implication in Pulmonary Arterial Hypertension

The NO pathway offers various molecular targets that can be exploited to help develop novel drugs to ameliorate the pathophysiological changes that occurs during PAH development. Some of the possible therapeutic targets offered by the NO pathway include arginase, ADMA, DDAH1, eNOS, PDE-5, cGMP, sGC and NO. This section highlights key drug molecules and target that focus mainly on the NO pathway and their implications in PAH.

L-arginine serves as a substrate not just for eNOS but arginase as well. This means an increase in arginase activity leads to a reduction in the availability of L-arginine to be converted to NO by eNOS (Pernow and Jung, 2013). Arginase metabolizes L-arginine to urea and L-ornithine (Luiking et al., 2012) as shown in Figure 1. Arginase exists in two isoforms namely arginase I and arginase II but arginase II has been found to be much more involved in the development of PAH in humans and animals (Jin et al., 2010; Cho et al., 2013 ). Deletion of arginase II in interleukin-13 overexpressing transgenic mice showed a significant decrease in medial wall thickness suggesting an important role of arginase II in PAH (Cho et al., 2013). PAH patients are known to have high arginase II activity compared to healthy controls with a consequent reduction in NO synthesis (Kao et al., 2015). A recent study found selective arginase II inhibitors L207-0525 and L327-0346 demonstrate a dose-dependent protective activity in monocrotaline-induced PAH rats. Furthermore, combining L207-0525 and L327-0346 with low dose (0.1 mg/kg) tadalafil produced a better protective effect in monocrotakine-induced PAH rats. Therefore, L207-0525 and L327-0346 are potential compounds needing further exploration for PAH treatment (Koklin and Danilenko, 2019).

Nitrates have shown promising signs in the amelioration of PAH in hypoxia- and monocrotaline-induced PAH animals. Both Intraperitoneal and nebulized sodium nitrite reduced pulmonary arterial pressure, right ventricular hypertrophy and vascular remodeling in MCT-induced PAH rats (Zuckerbraun et al., 2010; Pankey et al., 2012). Orally administered sodium nitrate have also shown protective effects in hypoxia-induced PAH mice (Baliga et al., 2012). However, a recent study found oral sodium nitrate does not substantially reduce established MCT-induced PAH in rats (Malikova et al., 2020). The different outcomes in the above studies could be attributed to the difference in disease severity in the PAH models as well as the routes and timing of drug administration (Malikova et al., 2020). Considering the poor response of certain patients to PDE5 inhibitors (PDE5i) due to reduced endogenous nitric oxide production, nitrates could serve as alternative sources of NO in such patients. Also, the impracticability of inhaled nitric oxide therapy further necessitates the need for alternate NO sources. More research has to be done to further explore the potential benefits of nitrates in PAH therapy especially in PDE5i-irresponsive patients.

Oxymatrine, an active alkaloid derived from the traditional Chinese herb *Sophora alopecuroides* was found to protect against hypoxia- and monocrotaline-induced PAH (Zhang et al., 2014). The mechanism behind the protective effect of Oxymatrine in monocrotaline-induced PAH is likely to be through the reduction of pulmonary ADMA levels even though it did not affect the level of DDAH1 (Dai et al., 2019).

Despite the fact that DDAH1 levels have been found to be reduced in hypoxia-induced PAH, it was uncertain how important of a role DDAH1 dysfunction plays in PAH. Recently, a study on a novel DDAH1 knockout (DDAH1^−/−^) rat strain model found DDAH1 dysfunction to significantly worsen RVSP and RVHI in DDAH1^−/−^ MCT model compared to the wild type MCT model (Wang et al., 2019). Although no *in vitro* experiment was carried out to target specific cells that are responsible for the progress of PAH, this study still shows how important DDAH1 dysfunction is in the development of PAH making it a possible PAH treatment target.

Apelin signaling is known to regulate endothelial NOS (eNOS) (Chandra et al., 2011). It has been discovered that the novel cyclic biased agonist of the apelin receptor, MM07 significantly reduces the elevation of right ventricular systolic pressure and hypertrophy induced by monocrotaline (Yang et al., 2019). They also found the elevation of eNOS and its mRNA as one of the mechanisms behind its protective effect in monocrotaline-induced PAH.

Udenafil, an oral phosphodiesterase-5 inhibitor approved for the treatment of erectile dysfunction has been found by a recent double-blind, placebo-controlled phase IIb clinical trial to improve 6-MWD in patients with PAH, especially those with a history of ERA therapy (Chang et al., 2019). The improvement in the 6-MWD with udenafil and ERA combination therapy group was found to be better than that of sildenafil and tadalafil in previous trials (Galiè et al., 2009a; Galiè et al., 2009b; Benza et al., 2018). Adverse side effects in the udenafil were also found to be mild and in the expected range (Chang et al., 2019).

TPN171, a new compound that inhibits PDE5 has been found to significantly reduce mPAP in a rat MCT-PAH model. Its efficacy was comparable to that of the sildenafil control group. It has a long half-life making once-daily dosing possible. The effective dose of TPN171 used in the animal experiment was 1 mg/kg which is lower than that of sildenafil (25 mg/kg). This property could make the occurrence of side-effects less likely if TPN171 is used in a clinical setting (Wang et al., 2019). TPN171 is currently in phase II clinical trial.

Evodiamine is a traditional Chinese medicine for the treatment of cancers (Dong et al., 2012). Evodiamine derivatives (S)-7e and (S)-7 days are newly discovered PDE5 inhibitors with very high selectivity (Zhang et al., 2020). Evodiamine derivative (S)-7 days significantly reduced mPAP and wall thickness in rat MCT-PAH model. Its efficacy is similar to that of sildenafil which was used as a positive control. Additionally, the study found a unique allosteric pocket of PDE5 using evodiamine derivative (S)-7e. Currently approved PDE5 inhibitors only bind to the substrate-binding pocket. This novel allosteric pocket regulates both enzymatic activity and pulmonary hemodynamic function of PDE5 thereby serving as a new therapeutic target for PAH treatment (Zhang et al., 2020).

A new study found PDE10 to be a novel therapeutic target for treating PAH. This study used a highly selective PDE10 inhibitor, 2b to explore the role of PDE10 in PAH. Compound 2b significantly reduced mPAP and RVHI in PAH rats making PDE10 a potential therapeutic target for PAH (Huang et al., 2019).

Studies have found natriuretic peptides to increase cGMP levels (Egom, 2015; Preston et al., 2016). Natriuretic peptides (NP) are inactivated by neprilysin. Natriuretic peptides are known to have antiproliferative effects on PASMCs and also attenuate the development of hypoxia-induced PH (Arjona at al., 1997; Zhao et al., 1999). Furthermore, the beneficial effect of sildenafil was found to be affected by natriuretic peptide activity in mice (Zhao et al., 2003). Also, the infusion of natriuretic peptides in the presence of sildenafil synergistically increased cGMP and reduced RVSP in hypoxia-induced PAH rats (Preston et al., 2016). A recent clinical trial demonstrated that the combination of a neprilysin inhibitor (racecadotril) with a PDE5 inhibitor (sildenafil or tadalafil) acutely increases NP and cGMP levels and improves pulmonary hemodynamics (Hobbs et al., 2019). This indicates that neprilysin inhibitors could have therapeutic use in PAH.

The mechanism of NO/cGMP-induced vasodilation is partly mediated by the activation of voltage-gated K^+^ (K_v_) channels (Cogolludo et al., 2001; Jackson, 2018). K_v_1.5 is particularly known to contribute the most to K_v_ current in PASMCs. K_v_7 channels have now been found to play a very significant role in generating K_v_ current in rat PASMCs (Mondéjar-Parreño et al., 2019). K_v_7 channel activation is now thought to be imperative to the electrophysiological and relaxant effects of NO donors and riociguat (Mondéjar-Parreño et al., 2019). This makes activation of K_v_7 channels a novel mechanism of action of vasodilators used in managing pulmonary arterial hypertension.

A new sGC stimulator, compound 13a (a pyrazolo [3,4-b] pyridine-3-yl pyrimidine derivative) has recently been found to exhibit similar *in vitro* vasorelaxation potential as riociguat on rat thoracic aorta rings and rat heart Langendorff preparation. Compound 13a also exhibited good oral bioavailability in male Beagle dogs which could make it a possible treatment candidate for PAH (Li et al., 2019). Compound 2 (a pyrazolo [3,4-b] pyridine derivative) is another novel sGC stimulator found to attenuate PAH (Hu et al., 2020). It reduced the migration of HPASMCs under hypoxic conditions significantly. Furthermore, it significantly improved RVSP, myocardial and vascular remodelling in hypoxia-induced PAH rats (Hu et al., 2020). We should notice that the PDE5 inhibitors block the breakdown of cGMP but these effects are dependent on NO availability and sGC activity. So the sGC stimulator or sGC activator may be effective in patients who have not sufficiently responded to a PDE5 inhibitor. Furthermore, sGC stimulator and sGC activator also show some difference, sGC stimulator acts on mature sGC and keep the activity of sGC, while sGC activator help to activate the damaged sGC.

## Conclusion

Great strides have been made in PAH research in recent years but searching for new therapeutical agents remains a big challenge in this field. Current therapy only slows disease progression but does not cure the disease. So far the current clinical practice has strongly suggested that targeting the NO pathway is the strategy with the most potential. Critical molecules in the NO pathway such as DDAH1, PDE10 and cGKI show potential for becoming the next new therapeutic targets in PAH treatment. Compounds or derivatives from plant extracts also have very bright prospect. Furthermore, medicines harboring the ability to enhance NO signal and other key signaling pathways at the same time exhibit better hope to finally cure this thorny disease.
